# Medical Items

**Published:** 1916-04

**Authors:** 


### MEDICAL ITEMS

### WHOOPING-COUGH A SERIOUS DISEASE

In an address before the New York Academy of Medicine, and reported in the Archives of Pediatrics, issue of August, 1914, John Lovett Morse, A. M., M. D. Professor of Pediatrics in the Harvard Medical School, made this significant statement: "The relative mortality from whooping-cough, scarlet fever and diphtheria is essentially the same throughout the country, whooping-cough being almost everywhere more fatal than scarlet fever and less fatal than diptheria * * * Instead of being a trifling affair, as it is usually considered to be by the laity, whooping-cough is a most serious and fatal disease. "Any disease which kills 10,000 children per annum is," as Rucker saqs, "a serious one." If bubonic plague were to kill that many children in the United States in one year, the whole world would quarantine against our country. A child dead of whooping-cough is just as dead as a child of plague.' "

In the same issue of the journal above referred to, the editor, an undoubted authority, says that "whooping-cough causes more deaths in children under one year than any other infectious disease."

In view of these startling facts, is it not just possible that the profession at large, like the average layman, has been too prone to look upon whooping-cough as an inevitable concomitant of childhood, and to underestimate its seriousness?

The Bordet-Gengou bacillus is recognized as the specific cause of whooping-cough, and the most rational method of treating the disease is by means of vaccine prepared from cultures of this bacillus. It is pertinent in this connection to refer to two such vaccines which are manufactured and marketed by Parke, Davis & Co. One bears the name of Pertussis Vaccine; the other is designated as Partussis Vaccine, Combined. The first-mentioned vaccine is indicated in cases diagnosed as pertussis, in suspected cases when a definite diagnosis is lacking, and as a prophylactic. The second is indicated in all cases of pertussis, but especially those which have persisted for some time, such infections being usually of the mixed type. The vaccines are administered hypodermically and are supplied in bulbs, in rubber-capped vials, and in glass syringes. The various packages are fully described in an announcement which appears elsewhere in this journal under the caption, "The Vaccine Treatment of Whooping-Cough." The advantages of the vaccine treatment are succinctly stated in the advertisement, which our readers are advised to consult.

---

### NOT A DIGESTIVE SUBSTITUTE

The amount of actual harm done with the best intention, by continually supplying the digestive organs with digestants, or ferments, instead of encouraging them to generate their own, is doubtless greater than we realize. It is not very often that one need order predigested food for a patient, although occasions may and do present themselves when this is advisable. But the indiscriminate use of pepsins and similar substances from the vegetable kingdom, in the management of many patients with weakened digestive powers, is scarcely to be justified. A much more useful remedy, because of its being a true stimulator to the digestive functions, gastric and intestinal, is Seng. This well-known preparation contains the active principles of Panax (Ginseng), and is especially useful because it stimulates and thus "helps them to help themselves"—obviously the most desirable therapeutics in all functional cases. It should be remembered, therefore, that Seng is not a ferment to digest food which weakened organs cannot care for in their natural manner. Instead, its action is to restore tone and vigor to the secretory structures so that they are able to evolve and supply their own ferments. Seng is a very agreeable remedy to take, and its benefits are manifested in surprisingly short order. In convalescence from fevers or disease impairing the digestive functions it is unquestionably one of the most efficient remedies being used by medical men today.

---

### THE PROPHYLACTIC IMPORTANCE OF EFFECTIVE CORRECTION OF LIVER DISORDERS

In connection with the modern tendency of medical practice to anticipate many human ills by instituting prophylactic treatment as soon as their possible occurrence is suspected—or, to perpetrate a bull, by "treating them before they begin"—it is especially interesting to note the growing recognition of the part played by the liver in the causation of many common affections. That the liver is an all important factor in the etiology of no small proportion of the metabolic disturbances, intestinal derangements and so-called auto-toxic disorders, is becoming more and more apparent as the physiologic functions of this great organ are given more careful attention and study. Moreover, as facts unfold, it is very evident not only that the importance of the liver has not been fully appreciated, but that prophylactic treatment to accomplish, with any degree of efficiency, the prevention of the ills referred to, must be directed primarily and principally to restoring and promoting the activity of the hepatic functions.

For many years the principal agents for attempting to restore the functional activity of the liver and regulate the portal circulation have been the hydragogue cathartics. In certain conditions these have been serviceable and more or less effective but in many others they have proven valueless and even harmful because of the exhaustion and depression resulting from the incidental catharsis.

In any comprehensive or effective scheme of prophylaxis of the affections due to insufficient or perverted hepatic activity the great desideratum is, therefore, to correct the liver condition without producing catharsis or purgation. The remedies that are able to meet this demand are very limited. In Chionia, however, the medical profession have a preparation of Chionanthus Virginica that can be relied upon to exert a promp stimulating and corrective effect on the liver without setting up a severe and drastic action of the bowels. The possibilities of such a product must at once be apparent. Certainly clinical experience has demonstrated its therapeutic utility, for under its use the functions of the liver are promptly restored to the normal, with all that this essentially means on metabolic processes in general, the elimination of toxic wastes and the regulation of the bowels. The use of Chionia, therefore, through its potent influence on the liver affords a dependable means of preventing many ills that all too often lead to serious and prolonged invalidism.

---

### WHEN THE STOMACH IS TIRED OR LAZY.

The artificial digestives such as pepsin, pancreative papain, etc., have their place in modern therapy, but they should always be used with care and common sense. How often do we encounter patients who are continually dosing themselves with pepsin or some one of the artificial digestives after each meal? Ninety-nine times out of a hundred this is unwise and a positive harm. Instead, the process of digestion should be encouraged—the stomach should be urged to do its own work—for any remedy that will specifically stimulate these functions to nearer normal action will produce permanent benefits that can never come from pepsin. Seng, is such a remedy, with a well defined secernent action on the glands and mucous membranes of the stomach that enables it to restore and increase the functional activity of an organ that in the great majority of instances is only over tired or indolent.

